# A plausible involvement of plasmalemmal voltage‐dependent anion channel 1 in the neurotoxicity of 15‐deoxy‐Δ^12,14^‐prostaglandin J_2_

**DOI:** 10.1002/brb3.1866

**Published:** 2020-11-16

**Authors:** Hiromi Koma, Yasuhiro Yamamoto, Noboru Okamura, Tatsurou Yagami

**Affiliations:** ^1^ Faculty of Pharmaceutical Sciences Himeji Dokkyo University Himeji Japan; ^2^ School of Pharmaceutical Sciences Mukogawa Women's University Nishinomiya Japan

**Keywords:** 15‐deoxy‐Δ^12,14^‐prostaglandin J_2_, anti‐VDAC antibody, caspase‐3, plasmalemmal voltage‐dependent anion channel, primary cortical neurons

## Abstract

**Introduction:**

15‐deoxy‐Δ^12,14^‐prostaglandin J_2_ (15d‐PGJ_2_) causes neuronal apoptosis independently of its nuclear receptor, peroxysome‐proliferator activated receptor γ. Its membrane receptor, chemoattractant receptor‐homologous molecule expressed on Th2 cells (CRTH2), did not also mediate the neurotoxicity of 15d‐PGJ_2_. In the present study, we ascertained whether membrane targets beside CRTH2 were involved in the neurotoxicity of 15d‐PGJ_2_.

**Methods:**

Neuronal membrane targets for 15d‐PGJ_2_ were separated by two‐dimensional electrophoresis, identified by proteomic approach. Their localizations were detected by microscopic immunofluorescence study. Cell viability and apoptosis was evaluated by MTT‐reducing activity and caspase‐3 activity, respectively.

**Results:**

Voltage‐dependent anion channel 1 (VDAC1) was identified as one of membrane targets for 15d‐PGJ_2_. Modification of VDAC1 with 15d‐PGJ_2_ was detected by pull‐down assay. VDAC1 was detected in the plasma membrane and localized on the neuronal cell surface. VDAC1 was partially colocalized with membrane targets for 15d‐PGJ_2_. The anti‐VDAC antibody significantly attenuated the neurotoxicity of 15d‐PGJ_2_, accompanied by the suppression of the 15d‐PGJ_2_‐stimulated caspase‐3.

**Conclusion:**

These findings suggested that the plasmalemmal VDAC might be involved in the neurotoxicity of 15d‐PGJ_2_.

## Graphical abstract

By proteomic analysis, we identified plasmalemmal voltage‐dependent anion channel (pl‐VDAC) as one of membrane targets for 15d‐PGJ_2_. An anti‐VDAC antibody suppressed the neurotoxicity of 15d‐PGJ_2_ partially, but significantly, suggesting that pl‐VDAC might contribute to the neurotoxicity of 15d‐PGJ_2_.

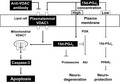

## 1 INTRODUCTION

15‐deoxy‐Δ^12,14^‐prostaglandin J_2_ (15d‐PGJ_2_) has been proposed as a mediator of neurodegenerative diseases including Alzheimer's disease (AD) (Yagami et al., 2017, 2018). Pathological features of AD are cortical atrophy, amyloid plaques, neurofibrillary tangles, and neuronal and synaptic loss in the brain including cerebral cortex (Selkoe, 1991). The precursor of 15d‐PGJ_2_, prostaglandin D_2_ (PGD_2_), formation was increased in the frontal cortex of AD patients as compared with those of controls (Iwamoto et al., 1989). Amyloid plaques are deposits of aggregated amyloid β (Aβ) peptides, which caused neuronal apoptosis via L‐type voltage‐dependent calcium channel in the primary neurons (Ueda et al., 1997). Prior to apoptosis, Aβ elevated the level of PGD_2_ transiently (Yagami et al., 2001).

PGD_2_ activates adenylate cyclase via Gs‐coupled DP1 (Hirata et al., 1994), which mediates neuroprotection (Yagami et al., 2018). On the other hand, it inactivates Gi‐coupled DP2, chemoattractant receptor‐homologous molecule expressed on Th2 cells (CRTH2) (Hirai et al., 2001). PGD_2_ is nonenzymatically metabolized to Δ^12^‐prostaglandin J_2_ (Δ^12^‐PGJ_2_) and 15d‐PGJ_2_ (Fitzpatrick & Wynalda, 1983; Shibata et al., 2002; Yagami et al., 2003). Although PGD_2_ appeared to induce neuronal apoptosis via 15d‐PGJ_2_ (Yagami et al., 2003), it has not yet been ascertained whether a DP2 (CRTH2) blocker inhibits the neurotoxicity of PGD_2_. A nuclear receptor of 15d‐PGJ_2_ is peroxysome‐proliferator activated receptor γ (PPARγ) (Forman et al., 1995; Kliewer et al., 1995), whereas its membrane receptor is CRTH2 (Hata et al., 2003; Sawyer et al., 2002). At low concentrations, 15d‐PGJ_2_ induces neuronal differentiation (Park et al., 2004) and neuroprotection (Koh et al., 2005) via PPARγ. In contrast, 15d‐PGJ_2_ exhibits neurotoxicity at high concentrations (Rohn et al., 2001; Yagami et al., 2001). 15d‐PGJ_2_ induced neuronal apoptosis independently of PPARγ (Yagami et al., 2003, 2018). On the other hand, it has not yet been ascertained whether CRTH2 is involved in the neurotoxicity of 15d‐PGJ_2_ or not.

Previously, we have reported 15d‐PGJ_2_‐modified proteins in neuronal plasma membrane (molecular weights more than 33 kDa, PI values less than 7) (Yamamoto et al., 2011). Furthermore, we have identified glycolytic enzymes, for example, neuron‐specific enolase (Yamamoto et al., 2015a), adaptor proteins, for example, 14‐3‐3ξ (Yamamoto et al., 2015b), and molecular chaperones, for example, HSP70 (Yamamoto et al., 2017) as targets for 15d‐PGJ_2_. However, we have not yet clarified how 15d‐PGJ_2_ caused neuronal apoptosis via the above three targets. Here, we analyzed other membrane proteins (molecular weights less than 40 kDa, PI values more than 7) and identified voltage‐dependent anion channel (VDAC) as one of membrane targets for 15d‐PGJ_2_. In mammals, VDACs are universal channels in all tissues (Shoshan‐Barmatz et al., 2010) and the most abundant protein in the mitochondrial outer membrane (Lemasters & Holmuhamedov, 2006). Mitochondrial VDAC contributes crucially to apoptosis in association with Bax/Bak and Bcl‐x_L_ (Shimizu et al., 2001). VDAC is extramitochondrially expressed in the plasma membrane (Dermietzel et al., 1994). Plasmalemmal VDAC (pl‐VDAC) also plays an important role in neuronal apoptosis in hippocampus (Akanda et al., 2008). In AD brains, VDAC is localized to caveolae and detected in dystrophic neurites of senile plaques (Ramirez et al., 2009). Our study demonstrated that pl‐VDAC was targeted for 15d‐PGJ_2_, suggesting that pl‐VDAC might contribute to the neurotoxicity of 15d‐PGJ_2_.

## 2 MATERIALS AND METHODS

### 2.1 Materials

Leibovitz's/L15 medium, trypsin, deoxyribonuclease I, fetal bovine serum, horse serum, penicillin, and streptomycin were obtained from Invitrogen. PGD_2_, Δ^12^‐PGJ_2_, 15d‐PGJ_2,_ and biotinylated 15d‐PGJ_2_ were obtained from Cayman Chemical. 3‐(4,5‐Dimethyl‐2‐thiazolyl)‐2,5‐diphenyl‐2H tetrazolium bromide (MTT) was purchased from DOJINDO. Immobiline™ DryStrip Gels (pH3‐10) and Amersham ECL Plus™ Western Blotting Detection Reagents were obtained from GE Healthcare Bio‐Sciences Corp. Sequence grade modified trypsin was purchased from Promega, and *N*‐(1‐pyrenyl) iodoacetamide was from Molecular Probes. Horseradish peroxidase‐linked antibody against biotin was obtained from Cell Signaling Technology. The protein concentration was measured using the BCA protein assay reagent obtained from Thermo Fisher Scientific. 1‐ethyl‐3‐(3‐dimethylaminopropyl) carbodiimide (EDC) was obtained from Pierce. Hoechst 33342 was purchased from Nacalai Tesque. All other chemicals were of reagent grade (Yamamoto et al., 2015b).

### 2.2 Animals

In macrolon cages maintained at 25°C on a 12‐hr light/dark cycle, pregnant female Wistar rats were housed individually with free access to food and water. All experimental procedures were approved by the Animal Care Committee of the Himeji Dokkyo University in accordance with NIH guidelines concerning the Care and Use of Laboratory Animals.

### 2.3 Primary culture of rat cortical neurons

Neuronal cell cultures were prepared from the cerebral cortex of day‐19 Sprague‐Dawley rat embryos as previously reported (Yagami et al., 2002). Our primary cultures contained approximately 95% neurons by immunostaining with an anti‐MAP2 antibody specific for neurons. Cerebral cortices were dissociated in isotonic buffer with 4 mg/ml trypsin and 0.4 mg/ml deoxyribonuclease I. 24 × well plates were used in the present study. Cells were plated at a density of 2.5 × 10^5^ cells/cm^2^ on poly‐l‐lysine‐coated dishes in conditioning medium, Leibovitz's/L15 medium supplemented with 5% FBS and 5% HS at 37°C in 5% CO_2_ and 9% O_2_.

### 2.4 Measurement of cell viability

As previously reported (Yagami et al., 2013), neuronal cell viabilities were evaluated by the MTT reduction assay reflecting activity of mitochondrial succinate dehydrogenase and morphologic criteria. To detect an involvement of pl‐VDAC on the neurotoxicity of 15d‐PGJ_2_, we used an anti‐VDAC antibody (sc‐32059), which can recognize VDAC1 and VDAC2. After various treatments, supernatants were replaced by 10 μM MTT in phosphate‐buffered saline. After incubation at 37°C for 2–3 hr, the resulting intracellular purple formazan was solubilized with DMSO and quantified by a CytoFluor^®^ Plate Reader at an excitation wavelength of 570 nm.

### 2.5 Preparation of neuronal plasma membrane‐enriched fraction

Cerebral cortices from rat brains were homogenized in 3 volumes of ice‐cold STEA solution (0.25 M sucrose, 5 mM Tris‐HCl [pH 7.5], 1 mM EGTA, and 50 karikllein units/ml aprotinin). The homogenate was filtered through three meshes and centrifuged at 700 *g* for 10 min. Fractions of nuclear and plasma membrane; The pellet containing nuclear and plasma membrane was resuspended in 120 ml of STEA solution by gentle homogenization, and the resuspension was dispersed in 1,080 ml of isosmotic Percoll solution (15.7% Percoll, 0.25 M sucrose, 1 mM EGTA, 50 karikllein units/ml aprotinin, and 10 mM Tris‐HCL [pH 7.5]). The second band from the surface in the supernatant was collected, washed by dilution with 2–3 volumes of HEA solution (1 mM EGTA, 50 karikllein units/ml aprotinin and 50 mM Hepes [pH 7.5]), and centrifuged at 10,000 *g* for 30 min. The pellet was suspended in HEA solution as the plasma membrane‐enriched fraction and stored in liquid nitrogen until used (Yamamoto et al., 2011).

### 2.6 Separation of 15d‐PGJ_2_‐targeted proteins by two‐dimensional electrophoresis

15d‐PGJ_2_‐targeted proteins were separated by two‐dimensional electrophoresis as previously reported (Yamamoto et al., 2011). The standard reaction mixture of 1 μM biotinylated 15d‐PGJ_2_ contained 50 mM Tris‐HCl buffer (pH 8.0), 100 mM NaCl, and plasma membranes (400 μg) in a total volume of 4 ml. Incubation was initiated by addition of the reaction mixture to plasma membranes and was carried out at 4°C for 24 hr in the presence or absence of unlabeled 15d‐PGJ_2_. We determined nonspecific binding by performing incubations with biotinylated 15d‐PGJ_2_ in the presence of 100 μM unlabeled 15d‐PGJ_2_. Two‐dimensional electrophoresis was performed with the CoolphoreStar Horizontal Gel Electrophoresis Unit IPG‐IEF (Anatech). The samples containing 400 μg of membrane lysates were dissolved in a rehydration buffer (5 M urea, 2 M thiourea, 2% [w/v] CHAPS, 2% [w/v] SB3‐10, 2% Pharmalytes, and 65 mM DTT) for the first dimensional isoelectric focusing (IEF). The pH range of the IEF was 3–10. Before IEF was performed, the gel strips were incubated with a swelling buffer (6 M urea, 2 M thiourea, 2% [w/v] TritonX‐100, 2% [w/v] SB3‐10, 2% Pharmalytes, 2.5 mM acetic acid, 0.0025% BPB, and 13 mM DTT). After IEF was performed, the gel strips were incubated with an SDS buffer (6 M urea, 32 mM DTT, 2% [w/v] SDS, 0.0025% BPB, 30% [v/v] glycerol, and 25 mM Tris‐HCl pH6.8) for 10 min and then with an alkylation buffer (6 M urea, 243 mM iodoacetamide, 2% [w/v] SDS, 0.0025% BPB, 30% [v/v] glycerol, and 25 mM Tris‐HCl pH6.8) for 10 min. For the second dimensional electrophoresis, polyacrylamide gel (12% acrylamide, 0.4% bis‐acrylamide, 10.6% glycerol, 0.1% SDS, 1.2% APS, 0.1% [v/v] TEMED, and 369 mM Tris‐HCl pH8.8) was used. All procedures followed the manufacturer's protocol. Separated proteins were then fixed in the gel using (a) 40% methanol and 10% acetic acid, (b) 10% methanol and 7% acetic acid, and (c) 10% methanol and 8% acetic acid. Then, they were stained with SYPRO Ruby protein gel stain and scanned using the FluoroPhoreStar^®^ 3000 (Anatech). The protein spots were visualized by Progenesis Same Spots (Nonliner Dynamics Ltd). For immunoblotting, gels were transferred to polyvinylidene fluoride membranes (Millipore Co.). The membranes were incubated with phosphate‐buffered saline containing 0.1% Tween20 (PBS/Tween) and 5% skim milk for blocking and washed with PBS/Tween. This procedure was followed by the addition of horseradish peroxidase‐conjugated anti‐biotin antibody and ECL reagents (GE Healthcare Bio‐Sciences). The spots were visualized by LAS‐3000 (Aisin Seiki Co., Ltd.).

### 2.7 Identification of 15d‐PGJ_2_‐targeted proteins by proteomic analysis

VDAC was identified as one of 15d‐PGJ_2_‐targeted proteins by proteomic analysis as previously reported (Yamamoto et al., 2011). Gel pieces were washed in 50 mM ammonium bicarbonic acid containing 50% acetonitrile for 10 min, twice. Then, they were dried in block incubator Bl‐516S (ASTEC Co., Ltd.) at 95°C for 10 min. Each sample was proteolytically degraded with 10 μl 1 mM ammonium bicarbonic acid containing 200 ng trypsin overnight at 37°C. The peptide in each gel was extracted with 50% acetonitrile containing 0.1% TFA followed by sonication for 15 min. The supernatant was collected, and peptides were further extracted with 75% acetonitrile containing 0.1% TFA followed by sonication for 15 min. Peptide extracts were concentrated to <10 μl using Speedvac concentrator. Then, they were desalted with Ziptip (Millipore Co.) and mixed with an equal volume of 5 mg α‐cyano‐4hydroxycinnamic acid (Shimadzu GLC Ltd.) dissolved in 0.5 ml 50% acetonitrile containing 0.1% TFA. One microliter samples were spotted onto a matrix assisted laser desorption/ionization (MALDI) plate. After air drying, spots were identified by MALDI time of flight mass spectrometry (MALDI‐TOF MS: Shimazu, AXIMA TOF^2TM^). MS spectra were collected over m/z 500–3,500. The acquisition parameters were Tunig mode: Reflectron, Mass range: 1–3,500, Max Laser Rep Rate: 10.0, CID: off, Power: 75, Profiles: 200, Shots: 5, Ion gate: Blank 900, P. Ext: 2,500, Scenario: Advanced, Profile average: All profiles, Peak width: 2 chans, Smoothing method: Gaussian, Smoothing filter width: 2 chans, Baseline filter width: 16 chans, Peak detection method: Thresh hold Apex, Thresh hold offset 0.500 mV, Use monoisotopic peak picking, Minimum mass 500, Maximam mass: 3,500, Resolution of the MS analyzer was 1,000 (0–1 kDa), 5,000 (1–2 kDa), and 10,000 (>2 kDa), Minimum isotope: 1, Maximum intensity variation: 90, and Overlapping distributions Minimum peak percent: 10. Proteins were identified with the MASCOT (Matrix Science) searching algorithms using the Swiss‐plot database. Probability‐based MOWSE scores were estimated by comparison of search results against estimated random match population and were reported as‐10* log 10(*p*), where *p* is the absolute probability. Scores greater than 61 were considered significant, meaning that for scores higher than 61 the probability that the match is a random event is lower than .05. The sequence version of the Swiss‐Prot was voltage‐dependent anion channel 1 (VDAC1). The interrogation parameters were Type of search: Peptide Mass Fingerprint, Enzyme: Trypsin, Fixed modifications: Variable modifications: Gln‐>pyro‐Glu (N‐term Q), Glu‐>pyro‐Glu (N‐term E), Oxidation (M), Mass values: Monoisotopic, Protein Mass: Unrestricted, Peptide Mass Tolerance: ±0.5 Da, Peptide Charge State: 1+ and Max Missed Cleavages: 1. Angiotensin II and ACTH were used as an internal standard. All protein identifications were in the expected size and PI range based on position in the gel.

### 2.8 Pull‐down assay and Western blotting

Pull‐down assay and Western blotting were principally performed as previously reported (Yamamoto et al., 2011). The standard reaction mixture of 1 μM biotinylated 15d‐PGJ_2_ contained 50 mM Tris‐HCl buffer (pH 8.0), 100 mM NaCl, and plasma membranes (400 μg) in a total volume of 4 ml. Incubation was initiated by addition of the reaction mixture to plasma membranes and was carried out at 4°C for 24 hr. Membrane lysates were incubated with streptavidin‐agarose beads (Invitrogen) at room temperature for 30 min. The beads were rinsed three times with lysis buffer. The proteins were eluted by boiling the beads in Laemmli sample buffer and analyzed by SDS‐PAGE followed by immunodetection with antibodies. A normal rabbit IgG ([sc‐2027] Santa Cruz) and an antibody to VDAC (rabbit polyclonal [#4866], Cell Signaling Technology) were used for immunodetection. The rabbit polyclonal antibody [#4866] is produced by immunizing animals with a synthetic peptide corresponding to the amino terminus of human VDAC‐1 (Cell signaling website).

### 2.9 Immunofluorescence microscopy

Microscopic immunofluorescence studies were performed as previously reported (Yamamoto et al., 2017). Neurons were primary cultured in glass bottom dishes (Nunc, Rochester) at a cell density of 20,000 cells/well. To target pl‐VDAC but not cytosolic ones, without permeabilization, neurons (DIV3) were immunostained with a normal rabbit IgG ([sc‐2027] Santa Cruz) and an anti‐VDAC1 antibody (rabbit polyclonal [AP1059], Calbiochem) (dilution 1:100) for 30 min at room temperature. The immunogen of rabbit polyclonal antibody [AP1059] is a synthetic peptide corresponding to amino acids surrounding Ser^104^ of human VDAC1. Following immunostaining, neuronal cultures were fixed with 3.7% formaldehyde in PBS for 15 min at room temperature. After two washing steps in PBS, Alexa Fluor 488‐conjugated anti‐rabbit IgG antibody was used as a secondary antibody. Alexa Fluor 488‐conjugated antibodies were excited at 490 nm and detected at 525 nm. Fluorescent signals were analyzed by confocal laser scanning microscopy (Carl Zeiss, LSM510). Magnification was 200. Number of fields was between 3 and 5. Cell numbers/field were between 100 and 200.

### 2.10 Fluorimetric assay of caspase‐3 activity

Caspases‐3 activity was assessed using a Caspase‐3 Assay Kit, Fluorimetric (SIGMA) as described previously (Yamamoto et al., 2011). Briefly, cells were seeded into 24‐well plates at a density of 2.5 × 10^5^ cells/cm^2^ followed by 24 hr incubation. After exposure to drugs for 24 hr, the supernatants were aspirated and cells were harvested with lysis buffer (50 mM HEPES, pH 7.4, 5 mM CHAPS and 5 mM DTT). The reaction buffer, including Acetyl‐Asp‐Glu‐Val‐Asp‐7‐amido‐4‐methylcoumarin (Ac‐DEVD‐AMC) and caspase‐3 specific substrates, was added to wells, and the production of AMC was sequentially detected in a CytoFluor^®^ Plate Reader at an excitation wavelength of 360 nm/emission 460 nm. Enzyme activities were determined as initial velocities expressed as nmol AMC min^−1^ ml^−1^. They were then corrected with the quantity of protein in each well detected by BCA protein assays (Thermo Fisher Scientific).

### 2.11 Statistical analysis

We analyzed the data statistically as previously reported (Yamamoto et al., 2017). Data are given as means ± *SD* (*n* = number of observations). At least, we performed three independent experiments on different days and confirmed their reproducibility. Data were analyzed statistically by use of Student's nonpaired *t* test for comparison with the control group, and data on various inhibitors and blocker groups were analyzed statistically by use of two‐way ANOVA followed by Dunnett's test for comparison with the PG‐treated group. We set *p* < .05 as statistically significant.

## 3 RESULTS

### 3.1 CRTH2 did not mediate neurotoxicities of PGD_2_ and 15d‐PGJ_2_

**FIGURE 1:**
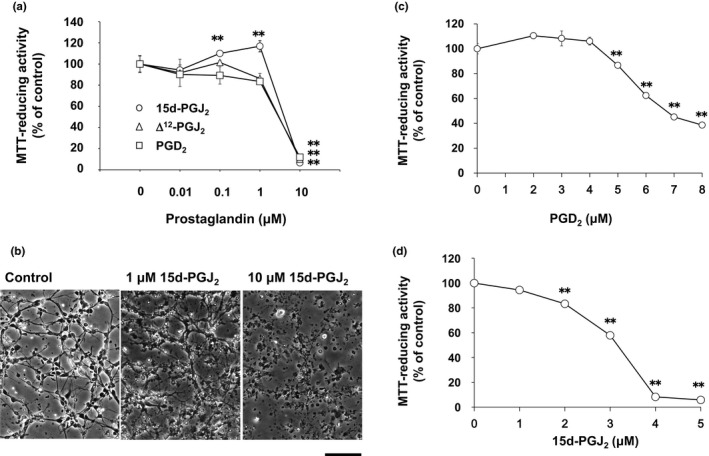
PGD_2_and its metabolites induced neuronal cell death. (a) Neurons (DIV2) were exposed to 15d‐PGJ_2_(cricles), Δ^12^‐PGJ_2_(triangles), and PGD_2_(squares) at concentrations between 0.01 and 10 μM for 50 hr. (b) Neuronal morphologies were photographed. Scale bar = 100 μm. (c) Neurons (DIV2) were exposed to PGD_2_at concentrations between 1 and 8 μM for 48 hr. (d) Neurons (DIV2) were exposed to 15d‐PGJ_2_at concentrations between 1 and 5 μM for 48 hr. MTT‐reducing activity was used to evaluate neuronal viabilities. Data points represent the mean ± *SD*of three experiments. **p < .01, versus control

We confirmed neurotoxicities of 15d‐PGJ_2_, Δ^12^‐PGJ_2,_ and PGD_2_ (Yagami et al., 2003). Interestingly, 15d‐PGJ_2_ increased the MTT‐reducing activity at the concentration from 0.1 to 1 μM (circles), whereas PGD_2_ (squares) and Δ^12^‐PGJ_2_ (triangles) did not (Figure 1a). To test the possibility that 15d‐PGJ_2_ might increase the cell number, we evaluated the cell number by the morphological criteria. Neurites were extended, whereas cell bodies were smooth and round in control cultures (Figure 1b). In contrast, neurites were shortened and fragmented, whereas neuronal cell bodies were shrunk and disrupted in 15d‐PGJ_2_‐treated cultures at 10 μM, resulting in the reduction of neuronal cell number (Figure 1b). However, we could not detect an increase of cell number in neurons treated with 1 μM 15d‐PGJ_2_ (Figure 1b).

**FIGURE 2:**
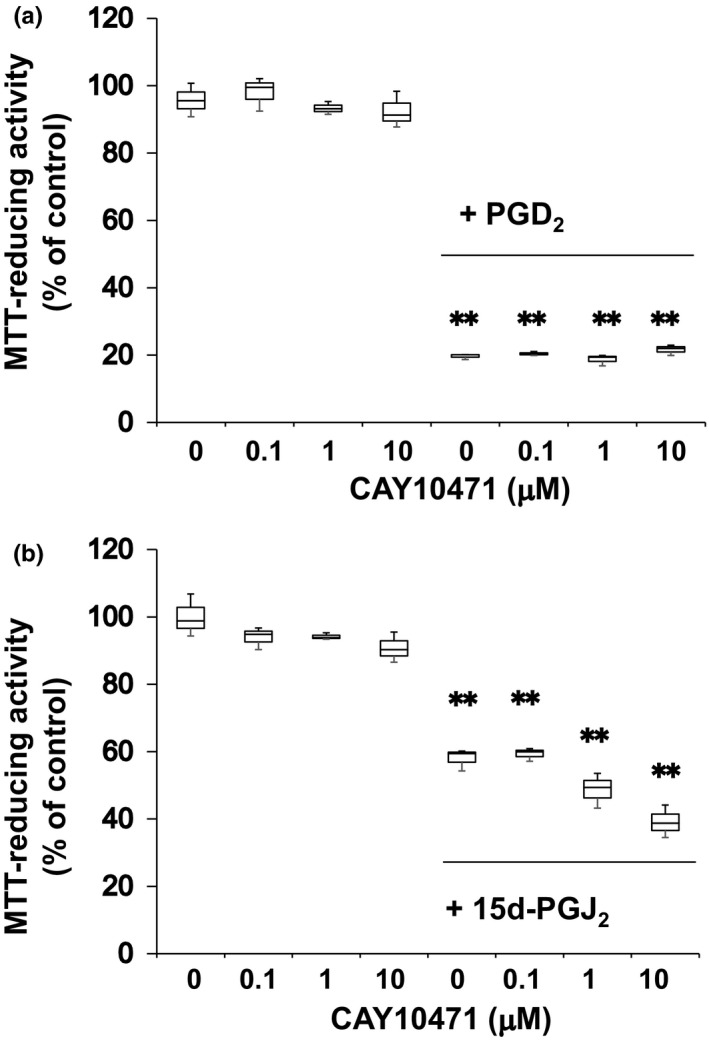
A CRTH2 antagonist did not suppress neurotoxicities of PGD_2_and 15d‐PG J_2_. (a) Neurons (DIV2) were exposed to CAY10471 at the indicated concentrations in the absence or presence of 7 μM PGD_2_for 48 hr. (b) Neurons (DIV2) were treated with CAY10471 at the indicated concentrations in the absence or presence of 3.5 μM 15d‐PGJ_2_for 48 hr. MTT‐reducing activity was used to evaluate neuronal viabilities. Data points represent the mean ± *SD*of three experiments. **p < .01, versus control

Δ^12^‐PGJ_2_ decreased the MTT‐reducing activity at 3 μM by 80%. Between 1 and 10 μM, we evaluated neurotoxicities of PGD_2_ and 15d‐PGJ_2_ in more detail. Half maximal neurotoxic concentration values of PGD_2_ and 15d‐PGJ_2_ were approximately 7 μM (Figure 1c) and 3.5 μM (Figure 1d), respectively. Since DP1 did not contribute to the neurotoxicity of PGD_2_ (Yagami et al., 2003), we ascertained whether PGD_2_ induced neuronal cell death via DP2/CRTH2 or not. A CRTH2 antagonist, CAY10471, did not affect the neuronal cell viability (Figure 2a,b). In addition, CAY10471 did not suppress the neurotoxicity of PGD_2_ (Figure 2a). Since CRTH2 is the common receptor for 15d‐PGJ_2_, Δ^12^‐PGJ_2,_ and PGD_2_ (Sawyer et al., 2002), we examined an effect of CRTH2 antagonist on the neurotoxicity of 15d‐PGJ_2_. CAY10471 did not prevent neurons from undergoing the 15d‐PGJ_2_‐induced cells death (Figure 2b). Thus, CRTH2 did not mediate the neurotoxicity of PGD_2_ and 15d‐PGJ_2_.

### 3.2 Isolation of membrane targets for 15d‐PGJ_2_

To isolate membrane targets for 15d‐PGJ_2_, biotinylated 15d‐PGJ_2_‐modified membrane proteins were separated by two‐dimensional electrophoresis. Biotinylated 15d‐PGJ_2_ was incubated with neuronal plasma membrane in the absence of serum to suppress nonspecific binding. The LC_50_ value of biotinylated 15d‐PGJ_2_ was almost 1 μM and similar to that of 15d‐PGJ_2_ (Yamamoto et al., 2011). As well as 15d‐PGJ_2_, biotinylated 15d‐PGJ_2_ also reduced the extension of neurites and shrank the cell body of neurons.

**FIGURE 3:**
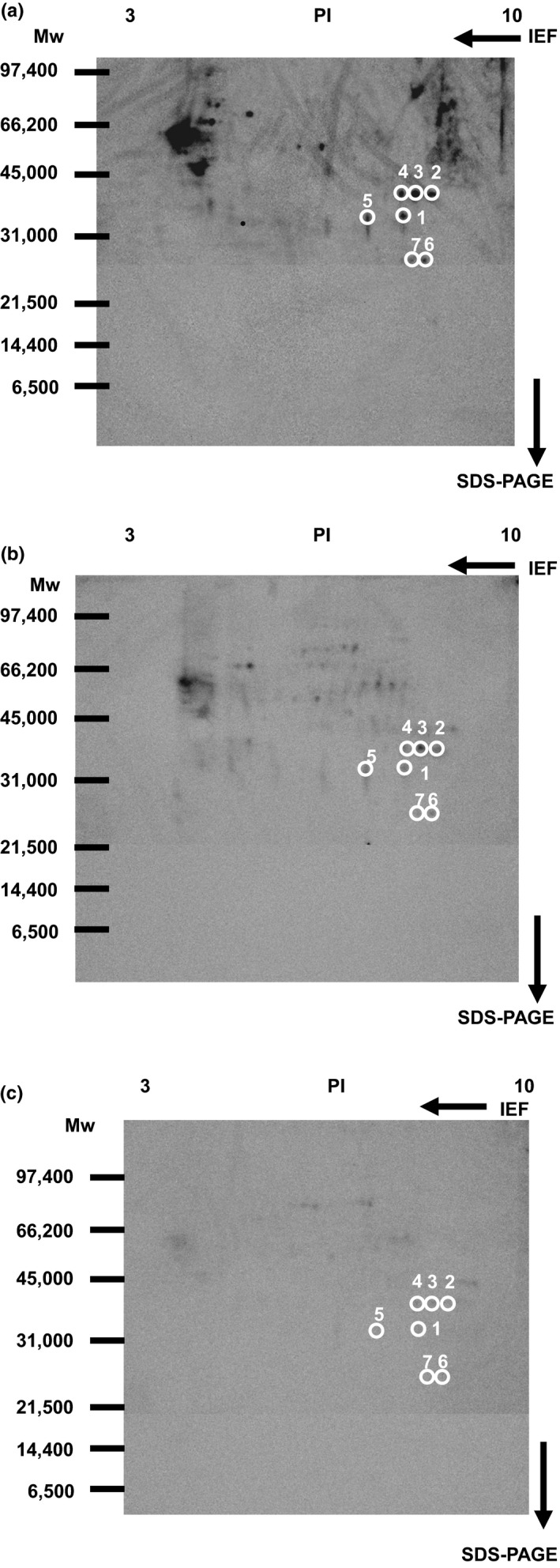
Western blotting of membrane proteins bound with 15d‐PGJ_2_‐biotin in the cortical neurons. Four hundred microgram membrane proteins were reacted with vehicle (a), 10 μM 15d‐PGJ_2_(b), or 100 μM 15d‐PGJ_2_(c) in the presence of 1 μM 15d‐PGJ_2_‐biotin. Two‐dimensional gel electrophoresis was used for separation of proteins from the plasma membrane‐enriched fraction by isoelectrofocusing (pH 10–3) and SDS‐PAGE. Spots surrounded by white circles were analyzed by MALDI‐TOF MS

Membrane proteins were labeled with 1 μM biotinylated 15d‐PGJ_2_ in the presence or absence of 10 or 100 μM 15d‐PGJ_2_. As the first step, biotinylated 15d‐PGJ_2_‐modified proteins were separated by isoelectric focusing electrophoresis. Next, membrane proteins at the same isoelectric points were separated by SDS‐PAGE. Western blotting of biotinylated 15d‐PGJ_2_‐protein conjugates was detected with anti‐biotin antibody‐HRP (Figure 3a). 15d‐PGJ_2_ decreased significantly the biotin‐positive spots at 10 μM (Figure 3b) and almost completely them at 100 μM (Figure 3c).

**FIGURE 4:**
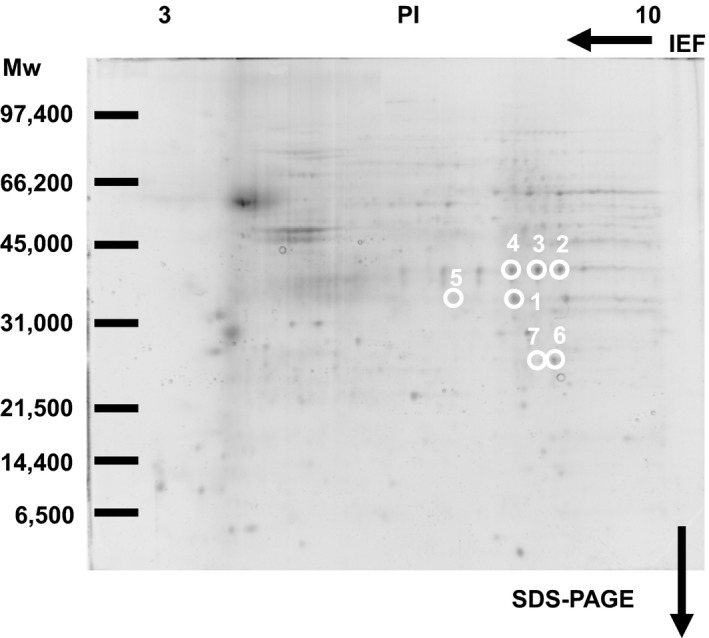
Membrane proteins stained by SYPRO Ruby. Four hundred microgram membrane proteins from the plasma membrane‐enriched fraction were reacted with 1 μM 15d‐PGJ_2_‐biotin and subsequently separated by two‐dimensional gel electrophoresis. Spots surrounded by white circles were analyzed by MALDI‐TOF MS

Previously, we have identified membrane proteins (molecular weights more than 33 kDa, PI values less than 7) as 15d‐PGJ_2_ targets (Yamamoto et al., 2011). Here, we selected seven spots to analyze membrane proteins (molecular weights less than 40 kDa, PI values more than 7) circled in Figure 3. Figure 4 indicated the patterns given by SYPRO Ruby fluorescence staining. When the two patterns were superimposed, there were seven SYPRO Ruby‐positive spots in accordance with biotin‐positive ones, which were surrounded with white circles in Figures 3 and 4.

### 3.3 VDAC was one of membrane targets for 15d‐PGJ_2_

**FIGURE 5:**
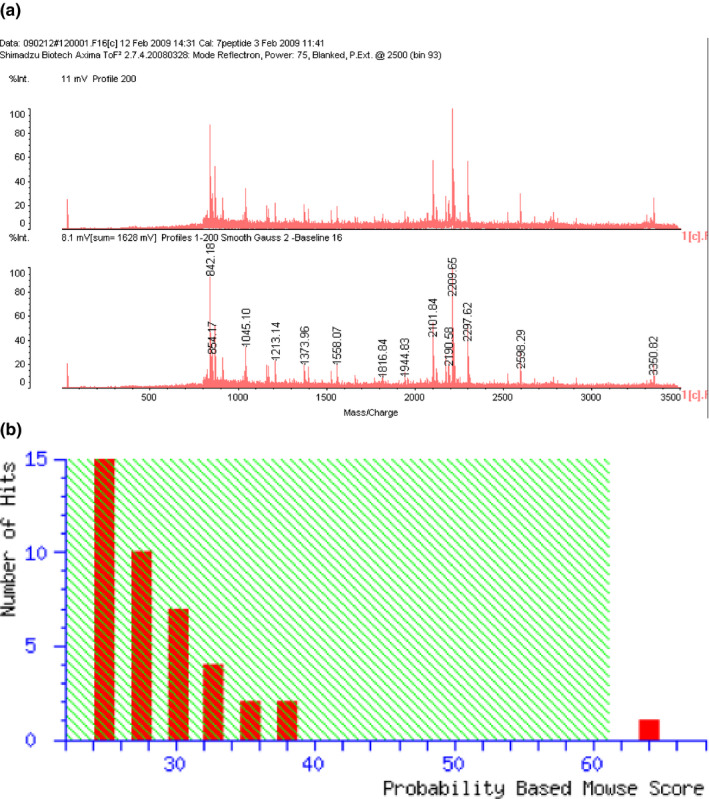
MS analysis of tryptic digests of membrane targets for 15d‐PGJ_2_. Following tryptic*in gel*digestion of spot #1 from Figure 4, digested peptides were analyzed by MALDI‐TOF MS. (a) Mass spectrum, (b) Probability‐based Mowse Score

**FIGURE 6:**
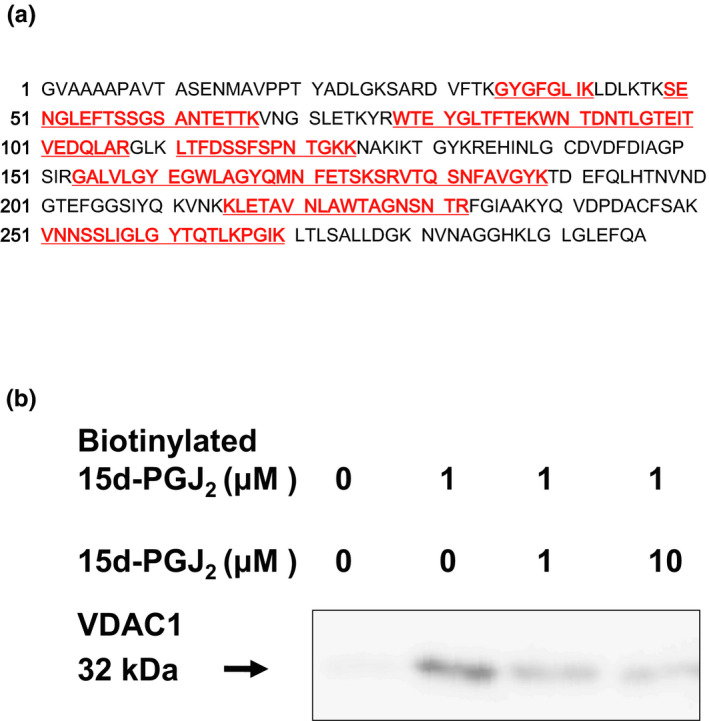
Pl‐VDAC was identified as one of membrane targets for 15d‐PGJ_2_. (a) Positions of matched peptides in the sequence of VDAC1. Matched peptides shown in red bold underlined. (b) Pull‐down assay: One micromolar biotinylated 15d‐PGJ_2_was reacted with cortical plasma membranes in the absence or presence of 1 or 10 μM 15d‐PGJ_2_. The reacted membranes were mixed with streptavidin beads. Biotinylated 15d‐PGJ_2_and its binding proteins were extracted and provided to SDS‐PAGE. Pl‐VDAC was detected by Western blotting with anti‐VDAC antibody

Seven circled proteins were excised from gels, digested by trypsin, and analyzed by MALDI‐TOF MS spectrometry (Figure 5a). Molecular weight and pI value of the spot #1 were 30–35 kDa and 8–9, respectively (Figures 3 and 4). Based on the peptide MS fingerprinting of the spot #1, it was predicted as VDAC1 (*Rattus norvegicus*) by MASCOT. Its probabilistic score adapted from MOWSE algorithm was 64 for VDAC1 (*p* < .05) (Figure 5b). Scores greater than 61 are considered significant, meaning that for the score 64 the probability that the match is a random event is lower than .05. Under the condition that peptide mass tolerance was ±0.5 Da, peaks in mass spectrum were assigned to 12 peptides (Figure 6a), which represents 48% sequence coverage (error ± 0.02%) in Figure 3c. The nominal molecular weight and the calculated pI value of VDAC1 were 32,060 Da and 8.35, respectively.

Next, spots around the spot #1 were analyzed by MASCOT. Although molecular weights of spots #2, #3, and #4 were same (35–40 kDa), their MOWSE scores less than 50 suggested that they might be glyceraldehyde‐3 phosphate dehydrogenase (Figures [Link], [Link], [Link]). A molecular weight of spot #5 was similar to the spot 1 (30–35 kDa), and its PI value from 7 to 8 was different from the spot #1 (Figure S4). Molecular weights of spots #6 and #7 were 25–30 kDa, and their PI values were from 8 to 9 (Figures S5 and S6). However, we could not succeed in identifying spots #5, #6, and #7 because of their low MOWSE scores.

### 3.4 Pl‐VDAC was detected in the nonpermeabilized neurons

To detect the interaction of 15d‐PGJ_2_ with VDAC, we performed the pull‐down assay with streptavidin‐agarose beads (Figure 6b). Membrane proteins were incubated with 15d‐PGJ_2_ at the indicated concentrations in the absence or presence of 1 μM biotinylated 15d‐PGJ_2_. Biotin‐labeled proteins were extracted by streptavidin‐agarose beads and separated by SDS‐PAGE. In the neuronal plasma membranes exposed to biotinylated 15d‐PGJ_2_, the 15d‐PGJ_2_‐target protein was detected in a position of 32 kDa protein by Western blotting with the anti‐VDAC antibody. Little was detected in those untreated with biotinylated 15d‐PGJ_2_. The biotinylated 15d‐PGJ_2_‐target adducts were displaced by 15d‐PGJ_2_ in a concentration‐dependent manner (Figure 6b). Thus, we confirmed VDAC as one of membrane targets for 15d‐PGJ_2_.

VDAC is also localized in the synaptic plasma membranes (Lim et al., 1984), suggesting the expression of neuronal cell surface. To avoid dyeing of mitochondrial VDAC, we performed staining procedures under nonpermeable condition. Neurons were incubated with the anti‐VDAC antibody, fixed, and treated with the secondary antibody, Alexa Fluor 488‐conjugated anti‐goat IgG antibody. The immunostaining with the anti‐VDAC antibody revealed a surface staining pattern, whereas that with control IgG did not (data not shown).

Next, we have performed double staining of neurons with the anti‐VDAC antibody (red) and the biotinylated 15d‐PGJ_2_ (green). Phase‐contrast photograph of neurons was indicated in the left upper panel of Figure S7a. Arrowed green fluorescent signals were selectively labeled by the biotinylated 15d‐PGJ_2_, but not stained by the anti‐VDAC antibody in the right upper panel. On the other hand, arrowed red fluorescent signals were selectively stained by the anti‐VDAC antibody, but not labeled by the biotinylated 15d‐PGJ_2_ in the left lower panel. Circled yellow fluorescent signals were merged signals of the anti‐VDAC antibody (red) and the biotinylated 15d‐PGJ_2_ (green) in the right lower panel. Almost half of the anti‐VDAC antibody‐stained signals were overlapped with the biotinylated 15d‐PGJ_2_‐stained ones (Figure S7a), whereas control IgG‐stained signals were hardly detected in neurons (Figure S7b). In addition, no fluorescent signal was detected in the “secondary antibody only controls” (Figure S7c). Thus, localization of VDAC and 15d‐PGJ_2_ targets was partially merged on the neuronal cell surface.

### 3.5 An anti‐VDAC antibody prevented neurons from undergoing 15d‐PGJ_2_‐induced cell death partially

**FIGURE 7:**
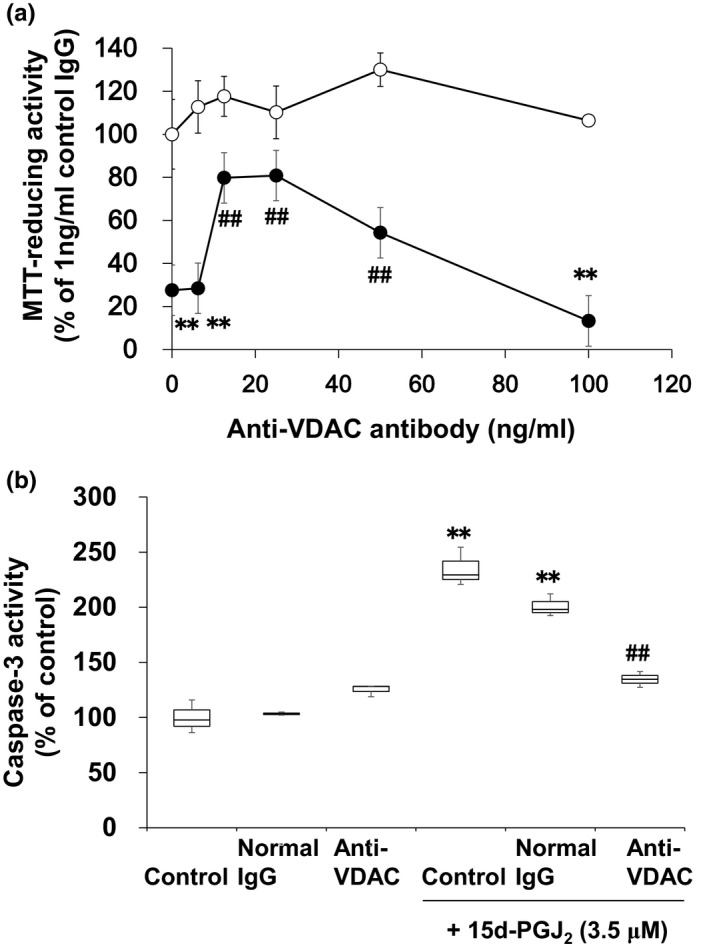
Effect of anti‐VDAC antibody on the 15d‐PG J_2_‐induced cell death. (a) Neurons (DIV2) were exposed to anti‐VDAC antibody at the indicated concentrations in the presence (closed circles) or absence (open circles) of 3.5 μM 15d‐PGJ_2_for 50 hr. The control for treatments were untreated cells. MTT‐reducing activity was used to evaluate neuronal viabilities. Data points represent the mean ± *SD*of three experiments. **p < .01, compared with control.^##^ *p* < .01, versus 15d‐PGJ_2_alone. (b) Effect of anti‐VDAC antibody on the 15d‐PGJ_2_‐activated neuronal caspase‐3. Neurons were exposed to 50 ng/ml normal IgG or anti‐VDAC antibody in the absence or presence of 3.5 μM 15d‐PGJ_2_for 48 hr. Caspase‐3 activity was determined by fluorimetric assay. The control for treatments were untreated cells. Data points represent the mean ± *SD*of three experiments. **p < .01, versus control.^##^ *p* < .01, versus 15d‐PGJ_2_alone

**FIGURE 8:**
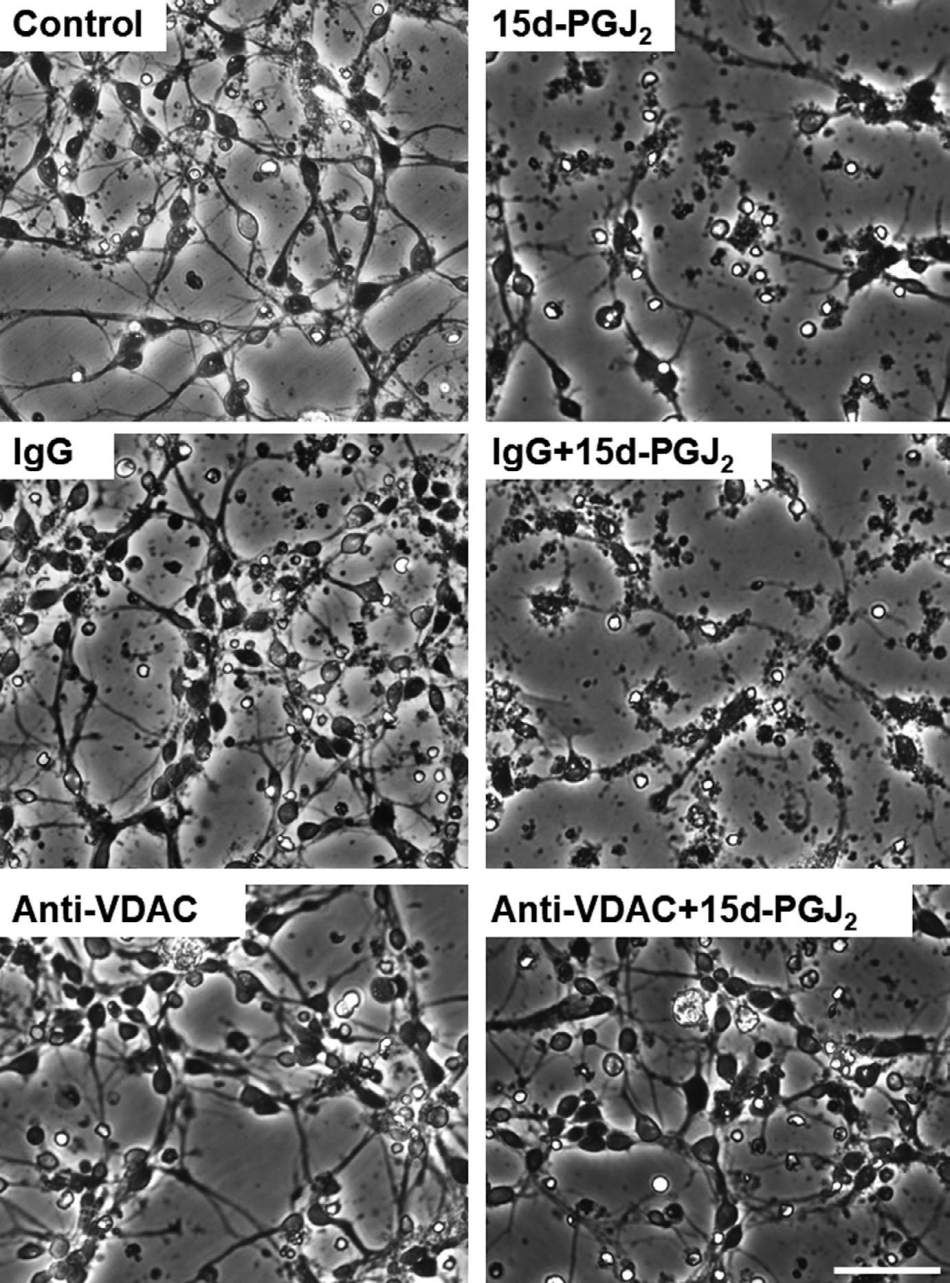
Effect of anti‐VDAC antibody on the 15d‐PGJ_2_‐degenerated neuronal morphology. Neurons were exposed to 50 ng/ml normal IgG or anti‐VDAC antibody in the absence or presence of 3.5 μM 15d‐PGJ_2_for 48 hr. Scale bar = 50 μm

Previously, we have reported that control IgG (normal goat IgG) did not exhibit neurotoxicity by itself (Yamamoto et al., 2017). The anti‐VDAC antibody did not also affect the MTT‐reducing activity of cortical neurons alone (Figure 7a). No neurotoxicity of control IgG and anti‐VDAC antibody was also detected by the morphological criteria (Figure 8). The anti‐VDAC antibody significantly suppressed the neurotoxicity of 15d‐PGJ_2_ at the concentration between 12.5 and 50 ng/ml. The anti‐VDAC antibody exhibited neuroprotective effect in a bell‐shaped fashion (Figure 7a). In addition, the anti‐VDAC antibody also prevented neurons from undergoing the 15d‐PGJ_2_‐degenerated morphology (Figure 8). On the other hand, normal IgG did not exhibit neuroprotective effect on the neurotoxicity of 15d‐PGJ_2_ (Figure 8). 15d‐PGJ_2_ induces neuronal apoptosis accompanied by activation of caspase‐3 (Koma et al., 2017; Rohn et al., 2001). We confirmed that 15d‐PGJ_2_ stimulated the caspase‐3 activity significantly (Figure 7b). The anti‐VDAC antibody restored the 15d‐PGJ_2_‐elevated level of caspase‐3 activity to the control level significantly, but normal IgG did not.

## 4 DISCUSSION

In the present study, we confirmed that 15d‐PGJ_2_ induces neuronal apoptosis at the high concentration (Rohn et al., 2001; Yagami et al., 2003). Previously, we have reported that its nuclear receptor, PPARγ, did not contribute to the 15d‐PGJ_2_‐induced neuronal cell death (Yagami et al., 2003). On the other hand, CRTH2 is the membrane receptor of PGD_2_ and 15d‐PGJ_2_ (Sawyer et al., 2002). However, a CRTH2 receptor antagonist did not attenuate neurotoxicities of PGD_2_ and 15d‐PGJ_2_. A selective CRTH2 receptor agonist 15d‐PGD_2_ did not exhibit neurotoxicity (Yagami et al., 2003). In addition, binding sites of PGD_2_ were not detected specifically in the neuronal plasma membranes (Yagami et al., 2003). CRTH2 is a seven‐transmembrane receptor, which couples with the pertussis toxin‐sensitive inhibitory GTP binding protein (Gi). However, pertussis toxin did not block the 15d‐PGJ_2_‐induced neuronal cell death (data not shown). Taken together, we ruled out the possibility that CRTH2 mediate the neurotoxicity of 15d‐PGJ_2_.

In the present study, 15d‐PGJ_2_ required several hours to induce neuronal cell death. However, it labeled membrane proteins on the neuronal cell surface within 1 hr. 15d‐PGJ_2_ possesses carboxyl group and acts as an anion under the physiological condition, indicating that 15d‐PGJ_2_ can not penetrate through the hydrophobic plasma membrane. Although transporters for prostaglandins including PGD_2_ have been reported (Lu et al., 1996), to our knowledge, no 15d‐PGJ_2_ transporter is reported. Although it has not yet been clarified how 15d‐PGJ_2_ is transported into cells, the present study suggested that 15d‐PGJ_2_ took several hours to be incorporated into neurons, activate caspase, inhibit proteasome, inactivate PI3K, and induce apoptosis after the binding with neuronal cell surface proteins.

Besides CRTH2, we have reported neuron‐specific enolase (Yamamoto et al., 2015a), 14‐3‐3ξ (Yamamoto et al., 2015b), and heat shock protein 70 (Yamamoto et al., 2017) among membrane targets for 15d‐PGJ_2_ (molecular weights more than 33 kDa, PI values less than 7) (Yamamoto et al., 2011). We are searching for evidences that 15d‐PGJ_2_ caused neuronal apoptosis via the above three targets. In the present study, we analyzed other membrane proteins (molecular weights less than 40 kDa, PI values more than 7). We identified VDAC as one of membrane targets for 15d‐PGJ_2_. Although VDAC is expressed on the outer membrane of mitochondria, Zhang et al. reported that its overexpression causes its mistargeting to the plasma membrane of the insulin secreting β cells (Zhang et al., 2019). On the other hand, VDAC is localized not only in mitochondria but also in plasma membrane of hippocampal neurons (Akanda et al., 2008). In hippocampal neurons, VDAC is neither overexpressed nor targeted to PM by mistake. Pl‐VDAC was localized on the cell surfaces of primary cortical neurons as well as hippocampal neurons (Akanda et al., 2008). Colocalization of VDAC with membrane targets for 15d‐PGJ_2_ was partial in the cortical neurons. Collectively, several pathways might be associated with the neurotoxicity of 15d‐PGJ_2_.

Does pl‐VDAC mediate the neurotoxicity of 15d‐PGJ_2_? In general, it is difficult to specifically target plasmalemmal proteins using molecular biological approaches without affecting the cytosolic ones. Anti‐VDAC antibodies inhibit pl‐VDAC and protect neurons from the staurosporine‐induced apoptosis (Akanda et al., 2008). Therefore, we also used the anti‐VDAC antibody to analyze specifically the function of pl‐VDAC, but not that of cytosolic one. Targeting of pl‐HSP70 with the anti‐HSP70 antibody triggered neuronal cell death. Hydrogen peroxide mediated the anti‐HSP70 antibody‐induced neuronal cell death, which did not require complement (Yamamoto et al., 2017). Furthermore, the anti‐HSP70 antibody suppressed the activity of caspase‐3 and the accumulation of ubiquitinated proteins (Yamamoto et al., 2017). In contrast, the anti‐VDAC antibody alone did not alter the viabilities of neurons as well as control IgG. However, it reduced the neurotoxicity of 15d‐PGJ_2_ partially, but significantly. Thus, we confirmed the protective effect of anti‐VDAC antibody on the neurotoxicity of 15d‐PGJ_2_ as well as that of staurosporine (Akanda et al., 2008).

15d‐PGJ_2_ exhibits contradictory functions in the brain. Neurites of PC12 cells are outgrown by nerve growth factor (NGF). At the low concentration, 15d‐PGJ_2_ promotes the NGF‐induced neurite outgrowth of PC12 cells, but PGD_2_ does not (Rohn et al., 2001; Satoh et al., 1999). Interestingly, the mitochondrial dehydrogenase was activated by 15d‐PGJ_2_ less than 2 μM, whereas the enzyme was not by PGD_2_ and Δ^12^‐PGJ_2_. There was no significant difference between neuronal cell numbers in the control culture and those in the 15d‐PGJ_2_‐treated one. 15d‐PGJ_2_ induces heme oxygenase 1 (HO‐1), which is an antioxidant enzyme via decreasing pro‐oxidant heme groups and increasing antioxidant molecules and carbon monoxide (Fujita et al., 2011). Therefore, the induction of antioxidant enzymes such as HO‐1 might contribute to the 15d‐PGJ_2_‐elevated MTT‐reducing activity at the low concentrations. Alternatively, we could not rule out the possibility that the number of mitochondria was increased or inhibition of other reactions using NADH was inhibited.

Anti‐VDAC antibodies have been reported to block pl‐VDAC and rescued neurons from apoptosis in the hippocampal neurons (Akanda et al., 2008). In the primary culture of cortical neurons, the anti‐VDAC antibody also exhibited the bell‐shaped neuroprotection against the neurotoxicity of 15d‐PGJ_2_. 15d‐PGJ_2_ activates caspase‐3 during neuronal apoptosis (Koma et al., 2017; Rohn et al., 2001). Neither control IgG nor the anti‐VDAC antibody affected the activity of caspase‐3. The anti‐VDAC antibody restored the caspase‐3 activity to the basal level. We confirmed that the anti‐VDAC antibody rescued neurons from the 15d‐PGJ_2_‐induced apoptosis in the primary cortical neurons. The neuroprotective anti‐VDAC antibody (sc‐32059) used in the present study is recommended for detection of VDAC2. To a lesser extent, it can also recognize VDAC1 of mouse, rat, and human origin by Western blotting. The cross‐reactivity of sc‐32059 with VDAC1 suggests that this anti‐VDAC antibody might exhibit its neuroprotective effect via the immunological cross‐reaction with VDAC1. Further studies are required to clear how the neuroprotective effect of anti‐VDAC antibody exhibits bell‐shaped dose response curve.

The present study suggested that 15d‐PGJ_2_ induced neuronal apoptosis via several pathways including pl‐VDAC. 15d‐PGJ_2_ causes neuropathology via inhibiting the ubiquitin‐proteasome pathway (Hirata et al., 1994; Yagami et al., 2018). Recently, we have reported that 15d‐PGJ_2_ exhibited neurotoxicity via down‐regulation of the PI3K signaling (Koma et al., 2017; Yagami et al., 2017). We have reported neuron‐specific enolase (Yamamoto et al., 2015a), 14‐3‐3ξ (Yamamoto et al., 2015b), and HSP70 (Yamamoto et al., 2017) as membrane targets of 15d‐PGJ_2_ (Yamamoto et al., 2011). As well as pl‐VDAC, these targets were localized on the cell surface of neurons. Although antibodies against neuron‐specific enolase, HSP70, and 14‐3‐3ξ induced neuronal cell death as well as 15d‐PGJ_2_ (Yamamoto et al., 2015a, 2015b, 2017), we have not yet succeeded in providing any evidences that 15d‐PGJ_2_ induces neuronal apoptosis via the three membrane targets. 15d‐PGJ_2_ induces neuronal apoptosis independently of CRTH2 and PPARγ, suggesting a plausible involvement of targets for 15d‐PGJ_2_ beside the two receptors. By proteomic analysis, VDAC1 was identified as one of membrane targets for 15d‐PGJ_2_. Pl‐VDAC was localized on the neuronal cell surface and partially colocalized with membrane targets of 15d‐PGJ_2_. The anti‐VDAC antibody suppressed the neurotoxicity of 15d‐PGJ_2_ partially, but significantly. The present study suggested that pl‐VDAC1 might contribute to the neurotoxicity of 15d‐PGJ_2_.

## CONFLICT OF INTEREST

There is no conflict of interest.

## AUTHOR CONTRIBUTIONS

HK designed this study, conducted statistical analysis, and acquired data. YY designed this study, conducted statistical analysis, acquired data, and wrote the draft of the manuscript. NO provided resources and conducted statistical analysis. TY conceived and supervised this study, conducted statistical analysis, and wrote and edited the draft of the manuscript.

### Peer Review

The peer review history for this article is available at https://publons.com/publon/10.1002/brb3.1866.

## Supporting information

Figure S1

Figure S2

Figure S3

Figure S4

Figure S5

Figure S6

Figure S7a

Figure S7b

Figure S7c

## Data Availability

Further information regarding resources, reagents, and data availability should be directed to the corresponding author (yagami@gm.himeji-du.ac.jp) and will be fulfilled upon reasonable request.
