# Supplementary material for: A plausible involvement of plasmalemmal voltage‐dependent anion channel 1 in the neurotoxicity of 15‐deoxy‐Δ12,14‐prostaglandin J2
Source: Brain Behav. 2020 Nov 16;10(12):e01866. doi: 10.1002/brb3.1866 (PMC7749624; doi:10.1002/brb3.1866)
Supplement: Supplementary file 1 — Figure S1 [file BRB3-10-e01866-s001.pdf]

Spot #2

(a)

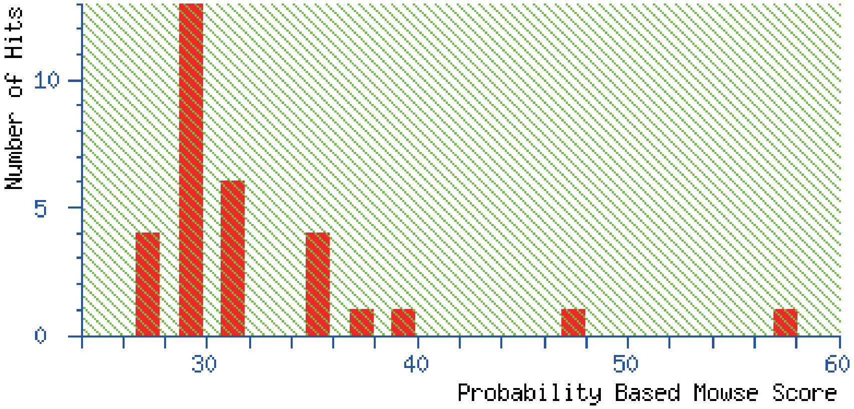

(b)

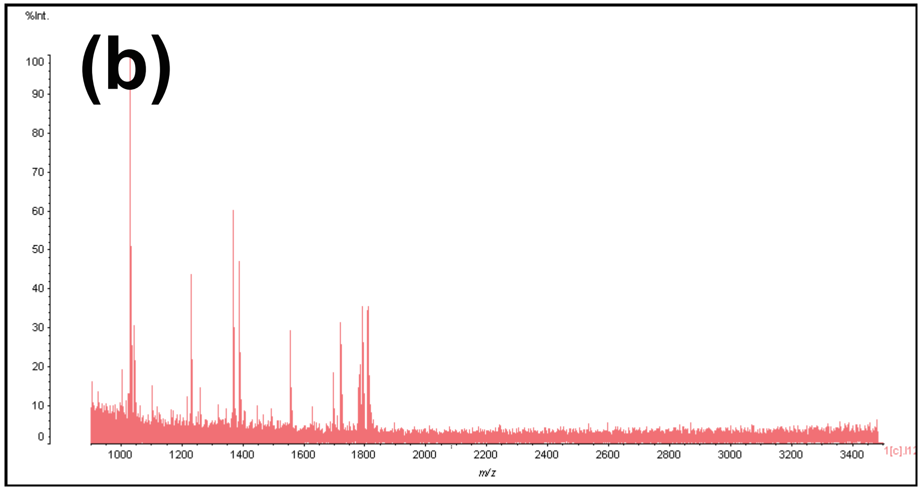

(c)

gi|8393418 **Mass:** 35805 **Score:** 58 **Expect:** 0.12 **Queries matched:** 7
glyceraldehyde-3-phosphate dehydrogenase [Rattus norvegicus]

gi|56188 **Mass:** 35813 **Score:** 58 **Expect:** 0.12 **Queries matched:** 7
glyceraldehyde 3-phosphate-dehydrogenase [Rattus norvegicus]

gi|109503241 **Mass:** 35800 **Score:** 58 **Expect:** 0.12 **Queries matched:** 7
PREDICTED: similar to glyceraldehyde-3-phosphate dehydrogenase isoform 2 [Rattus norvegicus]

gi|62653546 **Mass:** 35760 **Score:** 58 **Expect:** 0.12 **Queries matched:** 7
PREDICTED: similar to glyceraldehyde-3-phosphate dehydrogenase [Rattus norvegicus]

gi|56611127 **Mass:** 35771 **Score:** 58 **Expect:** 0.12 **Queries matched:** 7
Glyceraldehyde-3-phosphate dehydrogenase [Rattus norvegicus]
