# Supplementary material for: A plausible involvement of plasmalemmal voltage‐dependent anion channel 1 in the neurotoxicity of 15‐deoxy‐Δ12,14‐prostaglandin J2
Source: Brain Behav. 2020 Nov 16;10(12):e01866. doi: 10.1002/brb3.1866 (PMC7749624; doi:10.1002/brb3.1866)
Supplement: Supplementary file 3 — Figure S3 [file BRB3-10-e01866-s003.pdf]

# Spot #4

(a) (b)

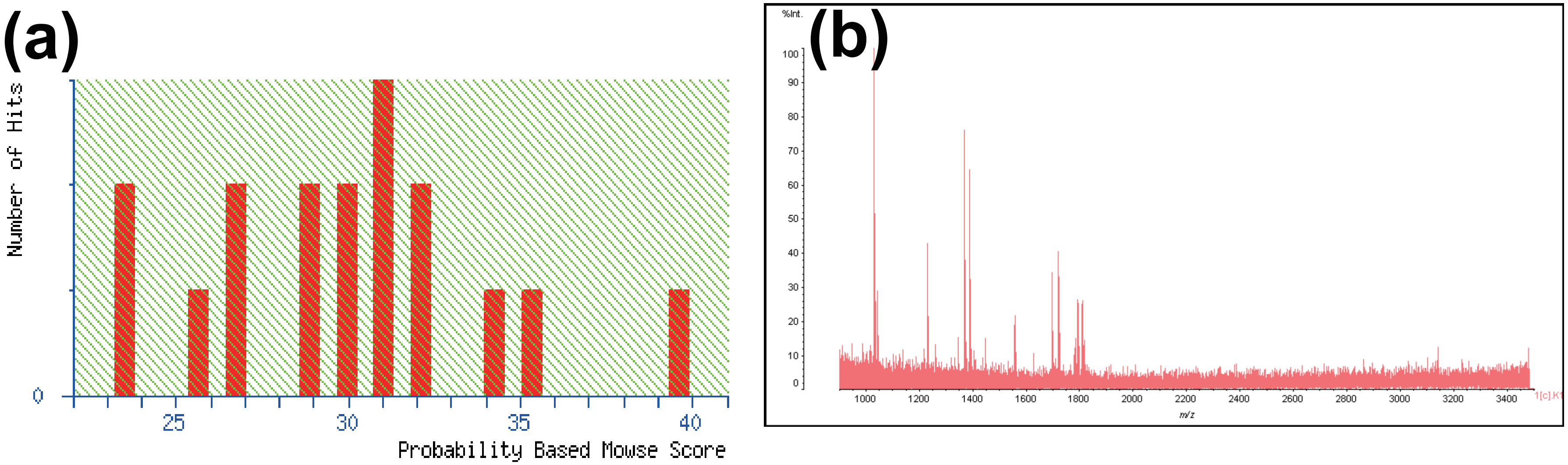


(c)

gi|8393418 **Mass:** 35805 **Score:** 40 **Expect:** 7.5 **Queries matched:** 4
glyceraldehyde-3-phosphate dehydrogenase [Rattus norvegicus]

gi|56188 **Mass:** 35813 **Score:** 40 **Expect:** 7.5 **Queries matched:** 4
glyceraldehyde 3-phosphate-dehydrogenase [Rattus norvegicus]

gi|109503241 **Mass:** 35800 **Score:** 40 **Expect:** 7.5 **Queries matched:** 4
PREDICTED: similar to glyceraldehyde-3-phosphate dehydrogenase isoform 2 [Rattus norvegicus]

gi|62653546 **Mass:** 35760 **Score:** 40 **Expect:** 7.5 **Queries matched:** 4
PREDICTED: similar to glyceraldehyde-3-phosphate dehydrogenase [Rattus norvegicus]

gi|56611127 **Mass:** 35771 **Score:** 40 **Expect:** 7.5 **Queries matched:** 4
Glyceraldehyde-3-phosphate dehydrogenase [Rattus norvegicus]
