# Supplementary material for: A plausible involvement of plasmalemmal voltage‐dependent anion channel 1 in the neurotoxicity of 15‐deoxy‐Δ12,14‐prostaglandin J2
Source: Brain Behav. 2020 Nov 16;10(12):e01866. doi: 10.1002/brb3.1866 (PMC7749624; doi:10.1002/brb3.1866)
Supplement: Supplementary file 4 — Figure S4 [file BRB3-10-e01866-s004.pdf]

Spot #5

(a)

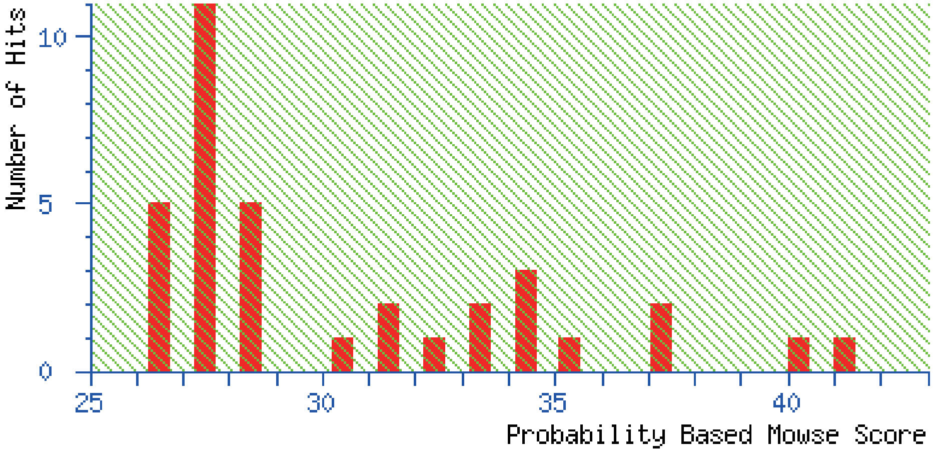

(b)

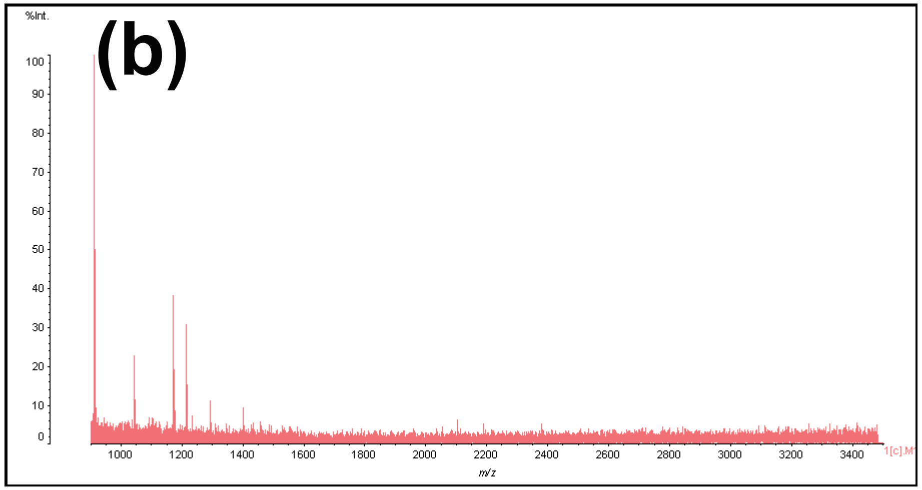

(c)

1. gi|149031250 **Mass:** 123556 **Score:** 41 **Expect:** 5.2 **Queries matched:** 10
vinculin (predicted), isoform CRA_a [Rattus norvegicus]
gi|157822133 **Mass:** 116542 **Score:** 30 **Expect:** 61 **Queries matched:** 9
vinculin [Rattus norvegicus]
gi|202581 **Mass:** 10613 **Score:** 29 **Expect:** 79 **Queries matched:** 3
alpha-1-inhibitor III precursor [Rattus norvegicus]

---

2. gi|197246004 **Mass:** 46431 **Score:** 40 **Expect:** 6.7 **Queries matched:** 6
Unknown (protein for MGC:189093) [Rattus norvegicus]

---

3. gi|203068 **Mass:** 71083 **Score:** 37 **Expect:** 13 **Queries matched:** 7
5-aminolevulinate synthase precursor
gi|42475958 **Mass:** 70975 **Score:** 30 **Expect:** 68 **Queries matched:** 6
aminolevulinate synthase H [Rattus norvegicus]
gi|202860 **Mass:** 70989 **Score:** 30 **Expect:** 68 **Queries matched:** 6
delta-aminolevulinate synthase precursor (EC 2.3.1.37)
