# Supplementary material for: A plausible involvement of plasmalemmal voltage‐dependent anion channel 1 in the neurotoxicity of 15‐deoxy‐Δ12,14‐prostaglandin J2
Source: Brain Behav. 2020 Nov 16;10(12):e01866. doi: 10.1002/brb3.1866 (PMC7749624; doi:10.1002/brb3.1866)
Supplement: Supplementary file 5 — Figure S5 [file BRB3-10-e01866-s005.pdf]

Spot #6

(a)

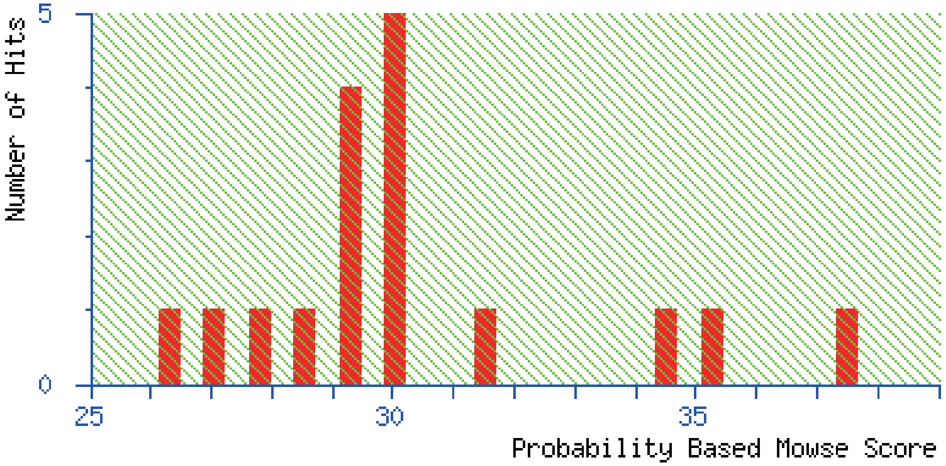

(b)

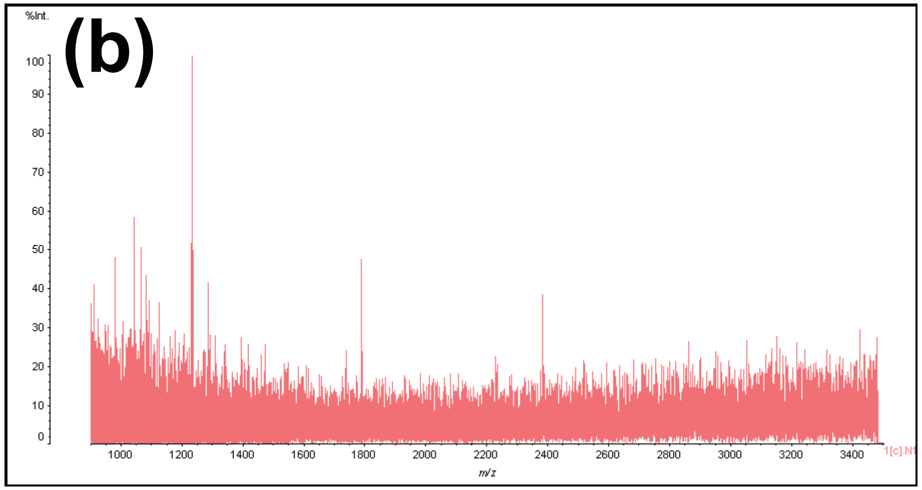

(c)

gi|109470857 **Mass:** 24783 **Score:** 38 **Expect:** 12 **Queries matched:** 4
PREDICTED: hypothetical protein [Rattus norvegicus]

gi|62667214 **Mass:** 55195 **Score:** 27 **Expect:** 1.4e+002 **Queries matched:** 4
PREDICTED: similar to cyclin D binding myb-like transcription factor 1 [Rattus norvegicus]

gi|149062275 **Mass:** 6396 **Score:** 30 **Expect:** 73 **Queries matched:** 2
rCG48501 [Rattus norvegicus]

gi|149050679 **Mass:** 14353 **Score:** 28 **Expect:** 99 **Queries matched:** 3
rCG61987 [Rattus norvegicus]

gi|149029909 **Mass:** 8073 **Score:** 27 **Expect:** 1.2e+002 **Queries matched:** 2
isocitrate dehydrogenase 3 (NAD), gamma, isoform CRA_e [Rattus norvegicus]

gi|112982914 **Mass:** 35788 **Score:** 26 **Expect:** 1.5e+002 **Queries matched:** 3
glycerophosphodiester phosphodiesterase domain containing 1 [Rattus norvegicus]
