# Supplementary material for: A plausible involvement of plasmalemmal voltage‐dependent anion channel 1 in the neurotoxicity of 15‐deoxy‐Δ12,14‐prostaglandin J2
Source: Brain Behav. 2020 Nov 16;10(12):e01866. doi: 10.1002/brb3.1866 (PMC7749624; doi:10.1002/brb3.1866)
Supplement: Supplementary file 6 — Figure S6 [file BRB3-10-e01866-s006.pdf]

Spot #7

(a)

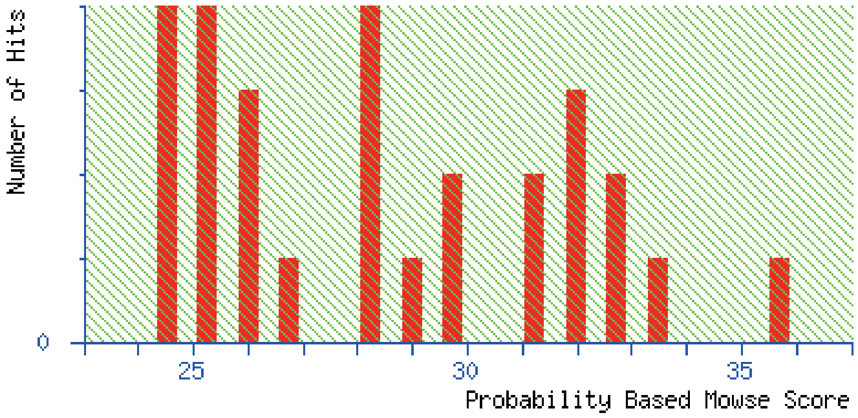

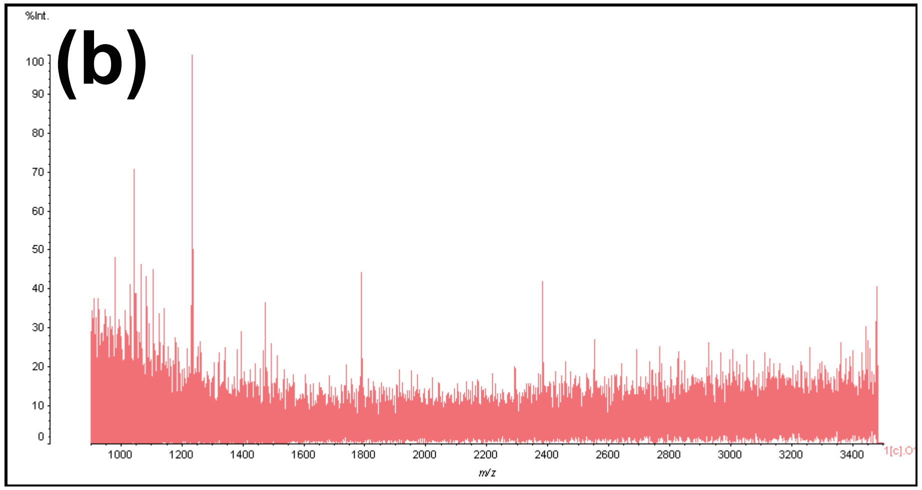

(c)

1. gi|149055329 **Mass:** 8758 **Score:** 36 **Expect:** 18 **Queries matched:** 5
rCG30539 [Rattus norvegicus]
gi|149031452 **Mass:** 2124 **Score:** 25 **Expect:** 2.2e+002 **Queries matched:** 2
rCG29135 [Rattus norvegicus]

---

2. gi|109476107 **Mass:** 35519 **Score:** 34 **Expect:** 30 **Queries matched:** 5
PREDICTED: similar to pre-mRNA splicing factor-like [Rattus norvegicus]

---

3. gi|149056211 **Mass:** 37994 **Score:** 33 **Expect:** 35 **Queries matched:** 5
LSM14 homolog A (SCD6, S. cerevisiae) (predicted) [Rattus norvegicus]
gi|189011632 **Mass:** 51196 **Score:** 28 **Expect:** 1.2e+002 **Queries matched:** 5
LSM14 homolog A [Rattus norvegicus]
