# Supplementary figures and images for: A plausible involvement of plasmalemmal voltage‐dependent anion channel 1 in the neurotoxicity of 15‐deoxy‐Δ12,14‐prostaglandin J2
Source: Brain Behav. 2020 Nov 16;10(12):e01866. doi: 10.1002/brb3.1866 (PMC7749624; doi:10.1002/brb3.1866)

**(a)**

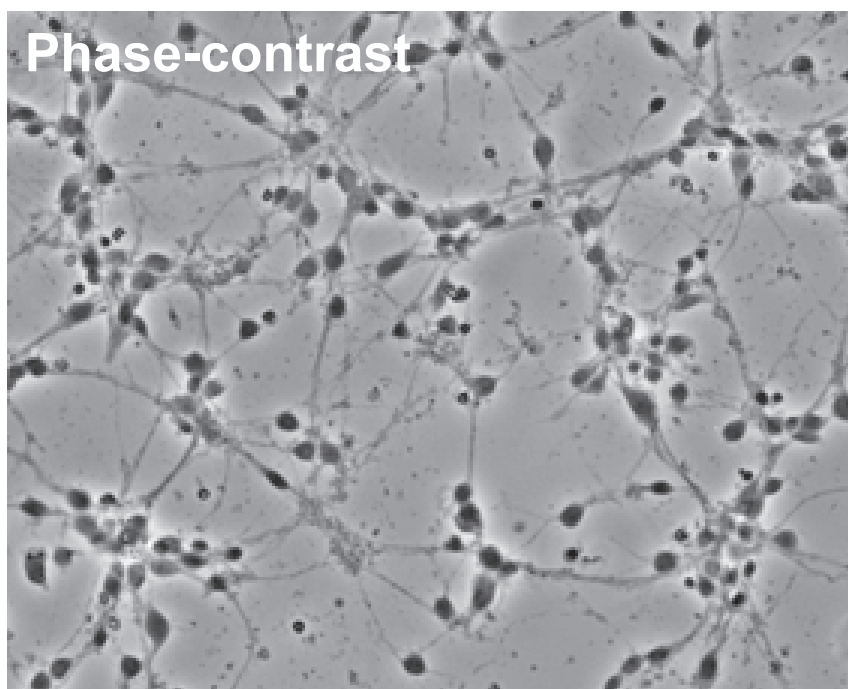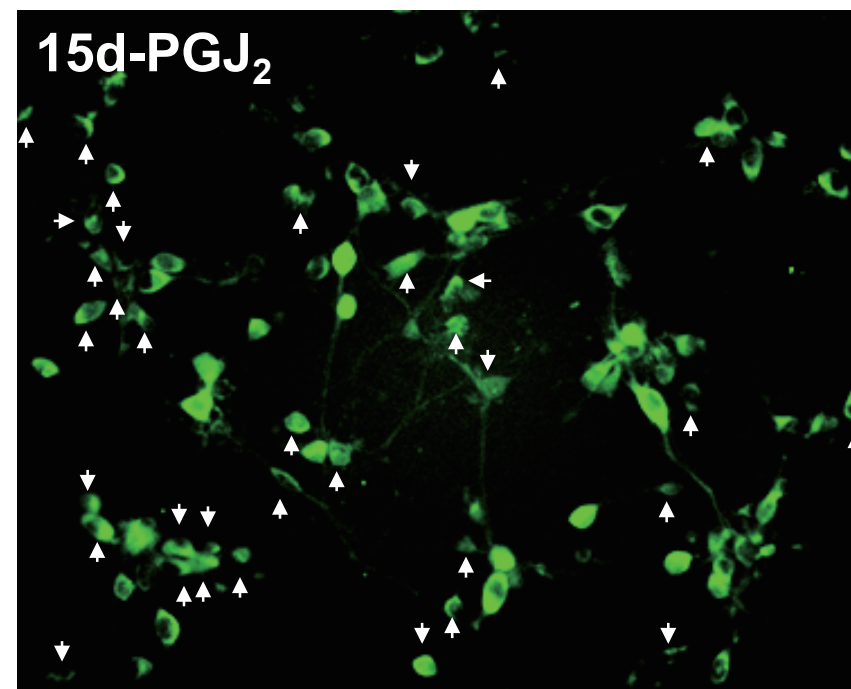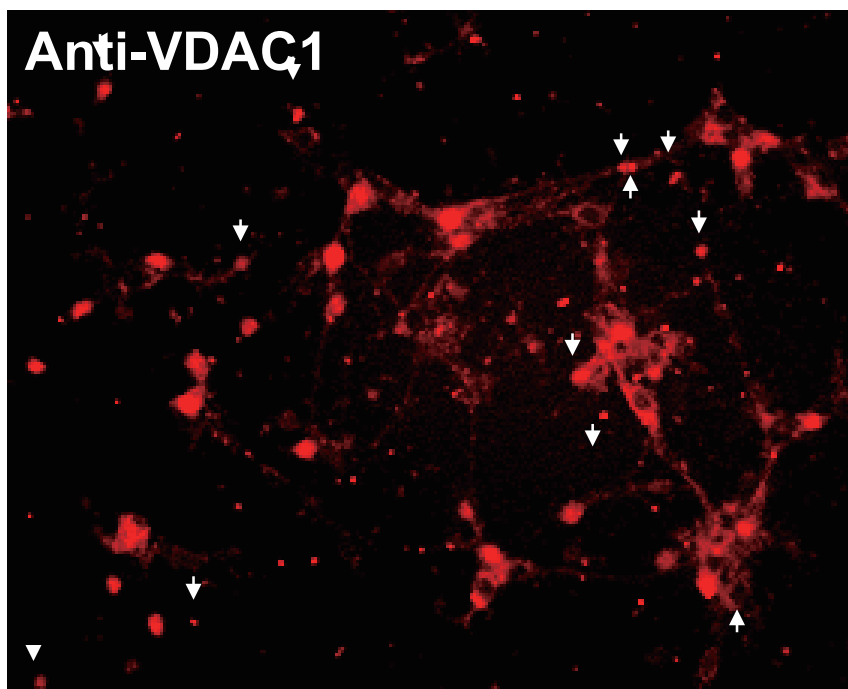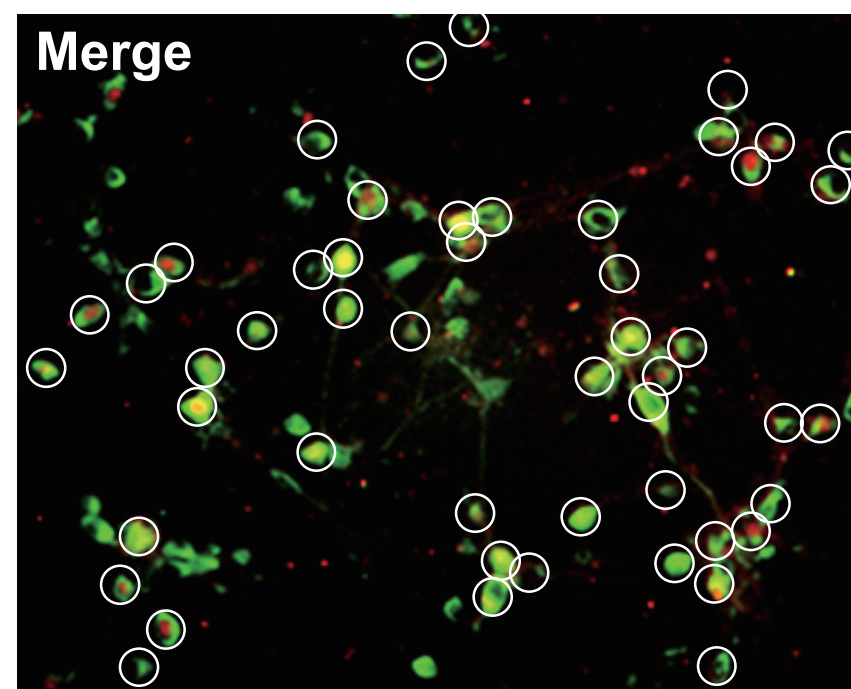

Supplement: Supplementary file 7 — Figure S7a [file BRB3-10-e01866-s007.pdf]

**(b)**

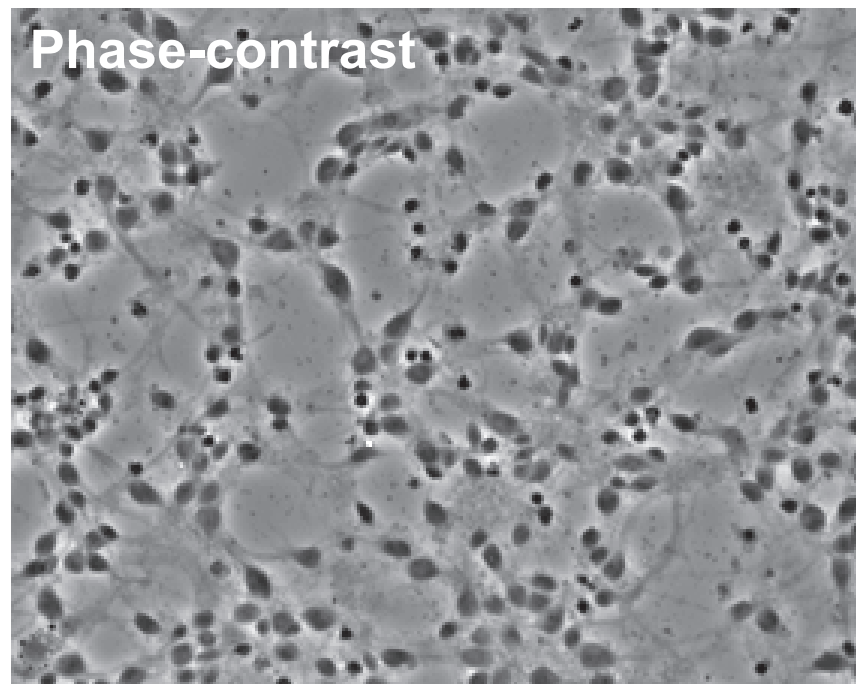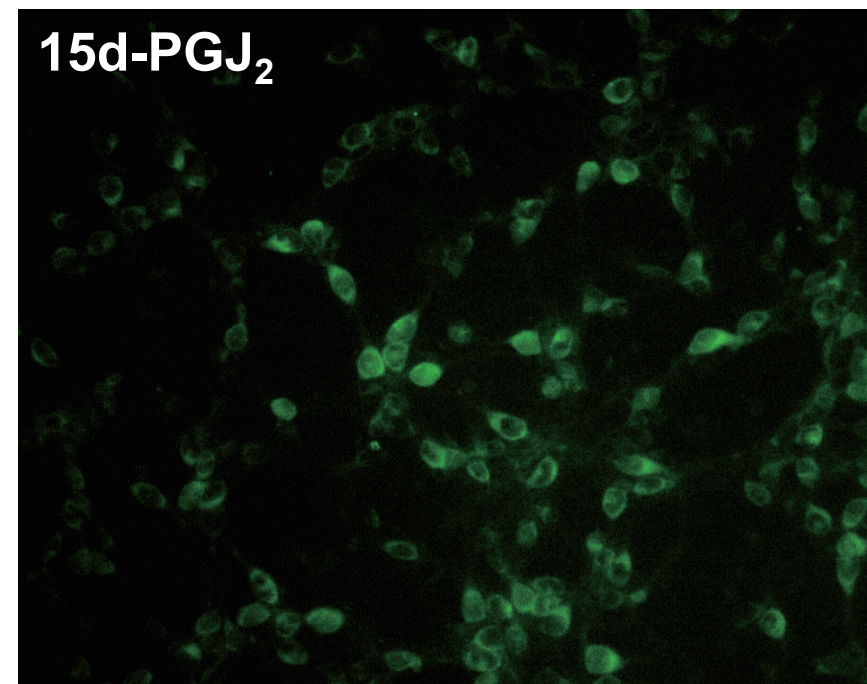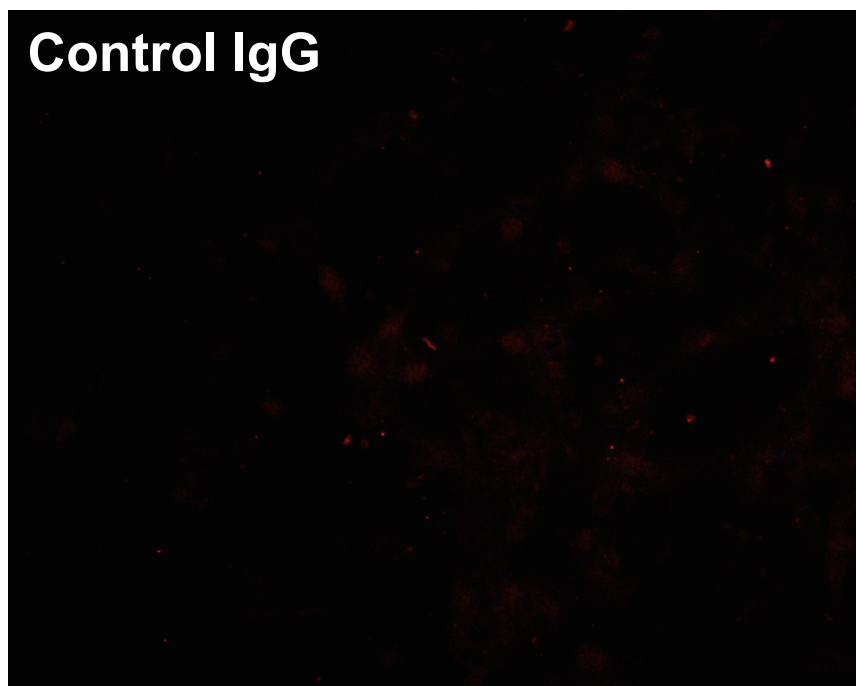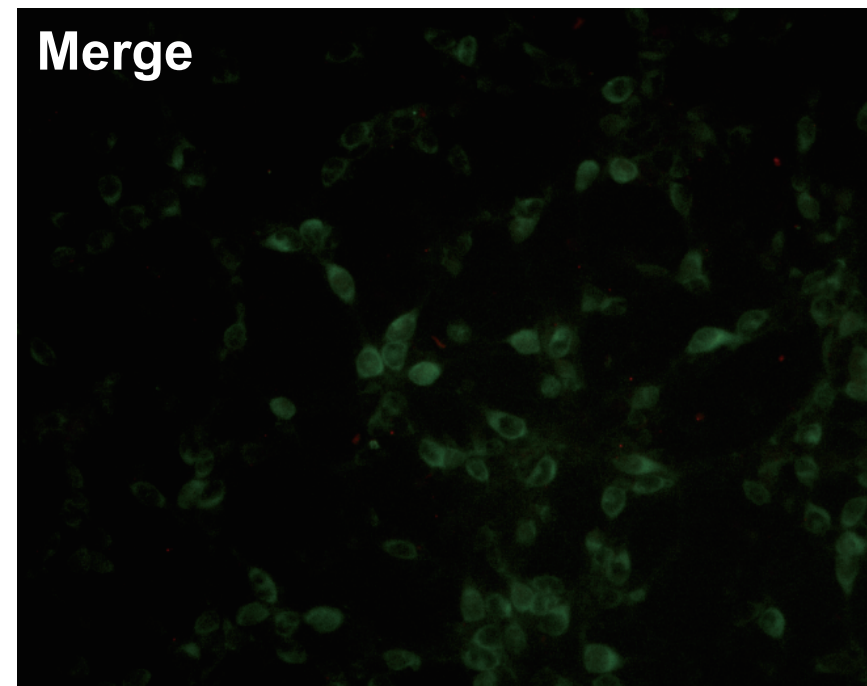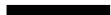

Supplement: Supplementary file 8 — Figure S7b [file BRB3-10-e01866-s008.pdf]

**(c)**

**Anti-Goat IgG antibody**

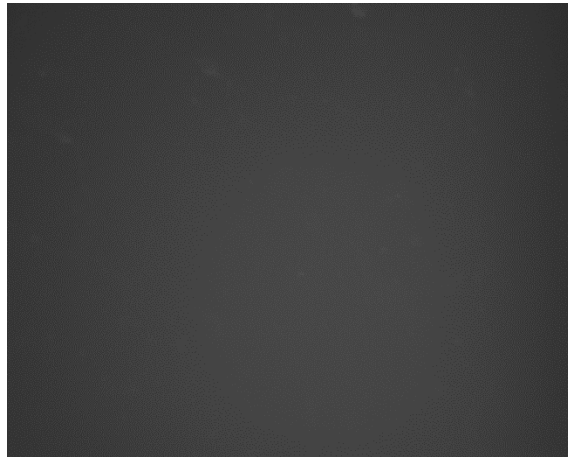

**Anti-rabbit IgG antibody**

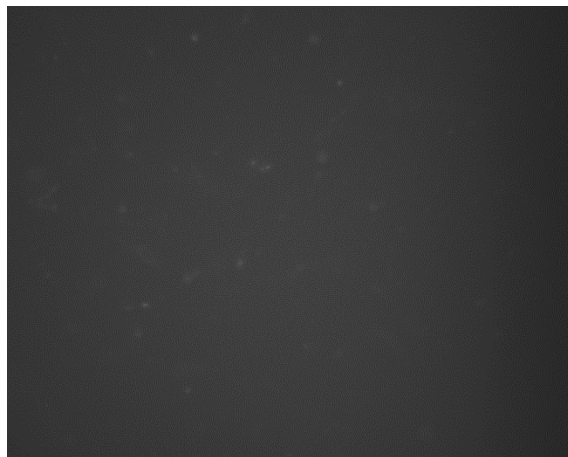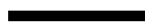

Supplement: Supplementary file 9 — Figure S7c [file BRB3-10-e01866-s009.pdf]
